# Blockade of advanced glycation end product formation attenuates bleomycin-induced pulmonary fibrosis in rats

**DOI:** 10.1186/1465-9921-10-55

**Published:** 2009-06-24

**Authors:** Lei Chen, Tao Wang, Xun Wang, Bei-Bei Sun, Ji-Qiong Li, Dai-Shun Liu, Shang-Fu Zhang, Lin Liu, Dan Xu, Ya-Juan Chen, Fu-Qiang Wen

**Affiliations:** 1 Division of Pulmonary Diseases, State Key Laboratory of Biotherapy of China, West China Hospital, West China School of Medicine, Sichuan University, Chengdu, Sichuan 610041, PR China; 2 Department of Respiratory Medicine, West China Hospital, West China School of Medicine, Sichuan University, Chengdu, Sichuan 610041, PR China; 3 Department of Pathology, West China Hospital, West China School of Medicine, Sichuan University, Chengdu, Sichuan 610041, PR China; 4 Department of Respiratory Medicine, the Third People's Hospital of Mianyang, Mianyang, Sichuan 621000, PR China

## Abstract

**Background:**

Advanced glycation end products (AGEs) have been proposed to be involved in pulmonary fibrosis, but its role in this process has not been fully understood. To investigate the role of AGE formation in pulmonary fibrosis, we used a bleomycin (BLM)-stimulated rat model treated with aminoguanidine (AG), a crosslink inhibitor of AGE formation.

**Methods:**

Rats were intratracheally instilled with BLM (5 mg/kg) and orally administered with AG (40, 80, 120 mg/kg) once daily for two weeks. AGEs level in lung tissue was determined by ELISA and pulmonary fibrosis was evaluated by Ashcroft score and hydroxyproline assay. The expression of heat shock protein 47 (HSP47), a collagen specific molecular chaperone, was measured with RT-PCR and Western blot. Moreover, TGFβ1 and its downstream Smad proteins were analyzed by Western blot.

**Results:**

AGEs level in rat lungs, as well as lung hydroxyproline content and Ashcroft score, was significantly enhanced by BLM stimulation, which was abrogated by AG treatment. BLM significantly increased the expression of HSP47 mRNA and protein in lung tissues, and AG treatment markedly decreased BLM-induced HSP47 expression in a dose-dependent manner (p < 0.05). In addition, AG dose-dependently downregulated BLM-stimulated overexpressions of TGFβ1, phosphorylated (p)-Smad2 and p-Smad3 protein in lung tissues.

**Conclusion:**

These findings suggest AGE formation may participate in the process of BLM-induced pulmonary fibrosis, and blockade of AGE formation by AG treatment attenuates BLM-induced pulmonary fibrosis in rats, which is implicated in inhibition of HSP47 expression and TGFβ/Smads signaling.

## Background

Pulmonary fibrosis is a devastating disorder and no effective treatment is available now. Although the underlying molecular mechanisms of pulmonary fibrosis remain not fully understood, increased synthesis and deposition of extracellular matrix (ECM) is confirmed to be an important pathological feature of pulmonary fibrosis [1]. Advanced glycation end products (AGEs), the irreversible products of nonenzymatic glycation of proteins, nucleic acids and lipids, are increased in situations with hyperglycemia and oxidative stress, which involves a series of complex biochemical events with oxidative and nonoxidative molecular rearrangements [2,3]. Previous studies have suggested that AGEs have multiple potential effects on various disorders [2-4]. T Matsuse et al reported AGE modified proteins accumulated in alveolar macrophages in patients with idiopathic pulmonary fibrosis [5], which suggests for the first time that AGEs probably contribute to the pathogenesis of pulmonary fibrosis. However, its role in pulmonary fibrosis has not been well-elucidated.

So far, several investigators have documented AGEs can induce ECM excessive deposition and expression of heat shock protein (HSP) 47 and profibrotic cytokines, such as transforming growth factor β (TGFβ)1 [6]. HSP47, a stress-inducible protein localized in the endoplasmic reticulum, is determined to play a specific role in the intracellular processing, folding, assembly and secretion of procollagens as a collagen molecular chaperone [7,8]. HSP47 expression is often prominent during the process of fibrosis in both humans and animal models [9-12]. In lung fibrosis, the HSP47-positive cells are considered to be the main source of collagen synthesis [9,13], which suggests a potentially important role of HSP47 in the pathogenesis of pulmonary fibrosis. TGFβ is a member of a large superfamily of pleiotropic cytokines which are involved in many biological activities, including cell proliferation, differentiation, migration and apoptosis [14]. Moreover, TGFβ, especially the isoform TGFβ1, is a key fibrotic stimulator in pulmonary fibrosis [15]. Generally, TGFβ performs its profibrotic effects via cascade stimulation of downstream intracellular Smad proteins. Among these Smads, Smad2 and Smad3 are necessary for TGFβ signal transduction [14,15]. Bleomycin (BLM), an antitumor drug, is often used to establish rodent models to mimic the pathologic features of idiopathic pulmonary fibrosis (IPF). Intratracheal instillation of bleomycin, induces pulmonary fibrosis following a gross inflammation in airways, which means a inflammatory and fibrotic phase is included in the process of BLM-induced lung injury. Time course studies have indicated the switch between the inflammatory and fibrotic phases is around day 9 after BLM treatment [16], and day 14 may be a more suitable time point for assessing lung fibrosis, considering the extensive fibrosis, but less variability in the fibrotic response and lower mortality than later time points [17]. Based on these points mentioned above, we used a rat model of pulmonary fibrosis stimulated by BLM instillation, treated with aminoguanidine (AG), an inhibitor of AGE formation by carbonyl-blocking [2], to explore whether AGE formation participates in BLM-induced pulmonary fibrosis, and whether it is involved in HSP47 expression and TGFβ signaling pathway.

## Methods

### Animals and Reagents

Pathogen free male Sprague-Dawley rats (250–300 g) were purchased from Experimental Animal Center of Sichuan University. Bleomycin was purchased from Harbin Bolai Pharmaceutical Co. Ltd (Harbin, China) and aminoguanidine was bought from Sigma (St. Louis, MO, USA).

### Treatment of Animals

**Figure 1:**
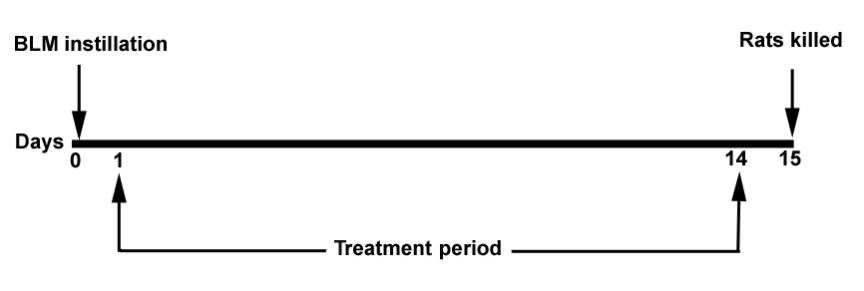
**Bleomycin administration and treatment protocol**. BLM instillation was performed on day 0. Following this, AG was administered by gavage from day 1 to day 14. All rats were killed on day 15.

This animal study was approved by the Panel on Laboratory Animal Care of West China School of Medicine, Sichuan University. These animals were housed in the temperature (22 ± 2°C) – and humidity (60 ± 5%)-controlled condition and kept on a 12-h light/dark cycle, with 24-h free access to the standard Purina (5001) rodent chow (autoclaved) and tap water that was heated to boiling for 20 min and then cooled to the room temperature before using it. Thirty rats were randomly divided into six experimental groups, with five rats per group, as follows: 1) Saline (SA)-treated with distilled water (DW) (SA group); 2) BLM-treated with DW (BLM group); 3) BLM-treated with AG (40, 80, 120 mg/kg) (BLM plus AG group); 4) SA-treated with AG (120 mg/kg) (AG group). Rats were anesthetized intraperitoneally with chloral hydrate (3 ml/kg) [18] and bleomycin (5 mg/kg) in 100 μl of saline was administered by intratracheal instillation with the same volume of saline in control animals. AG was dissolved in DW at a dose of 8 mg/ml. AG or DW was administered by gavage once daily from day 1 to day 14 after BLM or saline treatment (day 0) and all rats were sacrificed with exsanguination on day 15 (Figure 1).

### Histopathology

Middle lobes of right lungs were embedded in paraffin, following fixation in 10% buffering formalin, and then processed to obtain 4-μm sections for Masson's trichrome staining. Histopathologic evaluation of pulmonary fibrosis was performed using Ashcroft scoring method. Briefly, the grade of lung fibrosis was scored on a scale of 0 to 8 using the following criteria: grade 0, normal lung; grade 1 to 2, minimal fibrous thickening of alveolar or bronchiolar wall; grade 3 to 4, moderate thickening of walls without obvious damage to lung architecture; grade 5 to 6, increased fibrosis with definite damage to lung structure; grade 7 to 8; severe distortion of structure and large fibrous areas [19]. After the examination of 30 randomly chosen regions in each sample at a magnification of ×100, the mean score of all the fields was taken as the fibrosis score in each sample. The scoring method strictly followed the blind principle.

### Hydroxyproline Assay

To assess collagen accumulation, lung tissues (40 mg per rat lung, wet weight) were used for measurement of hydroxyproline content. Hydroxyproline assay was performed according to the instruction of hydroxyproline test kit from Nanjing Jiancheng Bioengineering Institute (Nanjing, China). In brief, frozen lung tissues were homogenized by a Polytron tissue homogenizer in saline containing 0.1 M phenylmethylsulfonylfluoride. The homogenized sample was hydrolyzed in 6 N HCl, and the hydroxyproline concentration was determined according to the method of Otsuka et al [20].

### RT-PCR

For RNA isolation, lung tissues were frozen in liquid nitrogen and stored in -80°C freezer immediately. Total RNA was extracted from frozen lung tissues (left lungs) using Trizol reagent (Gibco-BRL, Gaithersburg, MD, USA), and amplified using a PCR single-step kit (Promega, USA), according to the manufacturer's instructions. RT-PCR was performed with PTC-200 DNA Engine PCR cycler (MJ Research, Inc., USA). The primers, which were designed based on published sequence of these genes and synthesized by Invitrogen (Carlsbad, CA), as follows: HSP47, forward (5'-CAAGAA CA AG GC AG AC TTATCGC-3'); reverse (5'-TCTGAT T AT CTCGCACCAGGAAG-3'), β-actin, forward (5'-C C T C A TGAAGATCCTGACCG-3'); reverse (5'-ACCGCTCA TTGCCG ATA G TG-3'). β-actin served as the constitutive control. The annealing temperature for each primer pair was 59°C to HSP47 and 58°C to β-actin, respectively. The products were separated by agarose gel electrophoresis and visualized by Gelview (Bioteke Corporation, Beijing, China). Semiquantitative densitometric analysis was performed with the Bio-Rad Universal Hood and Bio-Rad Quantity One software (Bio-Rad, Hercules, CA). Means of the ratio of HSP47 band photodensity to β-actin band photodensity in various groups were presented.

### ELISA

Lung tissues for ELISA were homogenized in 10 mM Tris buffer (pH 7.4) containing 1% Triton X-100, 1 mM EDTA, 1 mM PMSF, 10 ug/ml aprotinin, and 10 ug/ml leupeptin. Protein concentration was quantitated by the Bicinchoninic Acid (BCA) Method according to the instruction of the BCA protein assay kit (Pierce, Rockford, IL). AGEs level in lung tissues was determined according to the instruction of the commercial ELISA kit (Uscnlife, Missouri City, TX). Samples were measured photometrically by an automated plate reader (Microplate Reader Model 1680; Bio-Rad, USA).

### Western Blot

Lung homogenates were prepared in lysis buffer, containing 50 mM Tris-HCl, 150 mM NaCl, 1% NP-40, 0.5% sodium deoxycholate, 2 mM NaF, 2 mM EDTA, 0.1% SDS and a protease inhibitor cocktail tablet (Roche Applied Science, Indianapolis, IN, USA). Protein concentration was quantitated by BCA Method described above. Equal amounts of protein samples (30 μg) from each group were loaded onto each lane of gels. Samples and prestained molecular weight markers (Bio-Rad, Hercules, CA) were subsequently electrophoresed on 12% Tris-glycine polyacrylamide gels and then were electrophoretically transferred onto polyvinylidene difluoride (PVDF) membranes (Millipore Corp., Marlborough, MA). The membranes were blocked for 1 h at room temperature with 5% Bovine Serum Albumin (BSA) and were incubated overnight at 4°C with primary antibodies including anti-HSP47 (Santa Cruz), anti-TGFβ1 (Cell Signaling), anti-Smad2 (Cell Signaling), anti-Smad3 (Cell Signaling), anti-p-Smad2 (Cell Signaling), anti-p-Smad3 (Cell Signaling), and anti-β-actin (Santa Cruz), at a dilution of 1:1000 in Tris-buffered saline with Tween-20 (TBST). β-actin served as the constitutive control to confirm equal amounts of protein loading. After washing with TBST, the membranes were incubated with the corresponding horseradish peroxidase-linked antirabbit antibody (Pierce, Rockford, IL) diluted 1:20000 in TBST for 1 h at room temperature. After further washing with TBST, immunoreactive bands were visualized by enhanced chemiluminescence (ECL), and quantified by densitometry with the Bio-Rad Universal Hood and Quantity One software (Bio-Rad). All results were normalized to β-actin levels in each lane.

### Statistical Analysis

All values were expressed as means ± standard deviation (SD). One-way ANOVA followed by Student-Newman-Keuls test was used to compare the differences among multiple groups. Significance was defined by a P value of 0.05. SPSS 13.0 software package (SPSS, Inc., Chicago, IL) was used for statistical analysis.

## Results

### AGEs level as well as bleomycin-induced pulmonary fibrosis is attenuated by AG treatment

**Figure 2:**
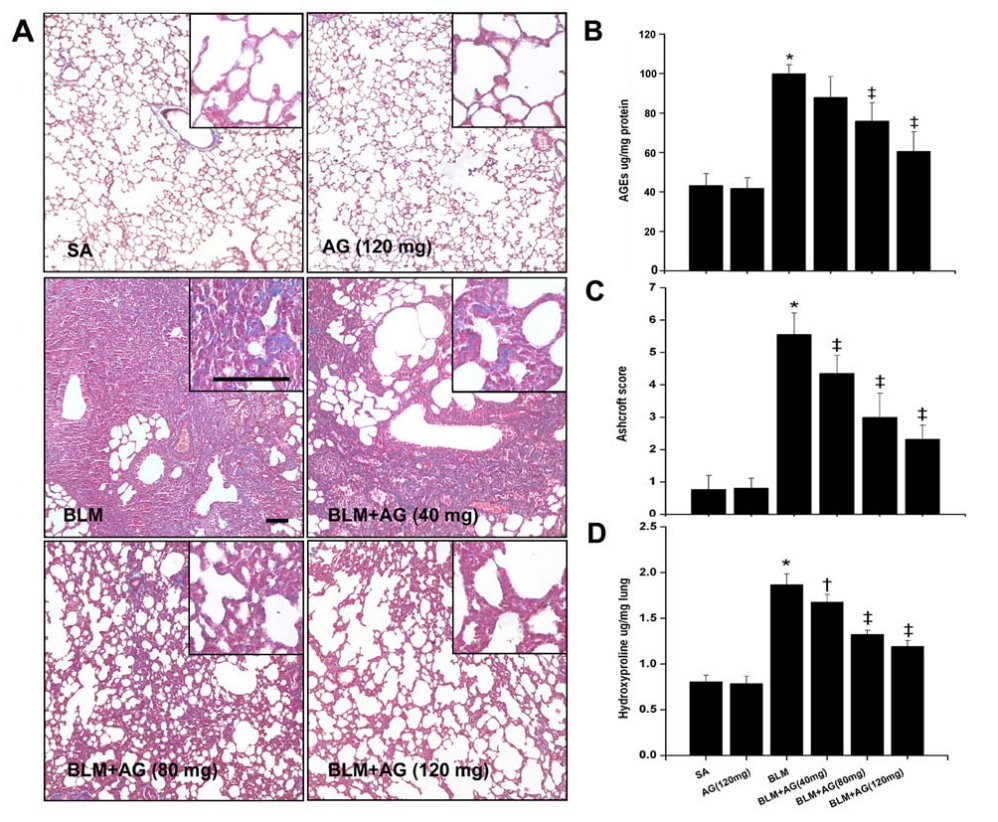
**Effect of AG on AGEs level and bleomycin-induced pulmonary fibrosis**. (A) Shown are representative photomicrographs of Masson-stained sections of lung tissues from each group. Collagens were stained blue. Bar = 100 μm. Comparisons of AGEs level (B), Ashcroft score (C) and lung hydroxyproline content (D) among different experimental groups were presented. Data represent mean ± SD, n = 5 in each group,* p < 0.01 vs SA group; † p < 0.05 vs BLM group; ‡ p < 0.01 vs BLM group.

Bleomycin instillation significantly induced pulmonary fibrosis (Figure 2A). Compared with the SA group, AGEs level in lung tissues was markedly increased in the BLM group (p < 0.01; Figure 2B), and was dose-dependently decreased with AG treatment, similar to the changes of Ashcroft score and lung hydroxyproline content (Figure 2C, D), which were used for assessing the degree of pulmonary fibrosis. Masson staining of lung specimens demonstrated that bleomycin instillation induced severe distortion of lung structure and accumulation of collagen fiber (blue) in rat lungs, whereas a well-alveolized normal histology was seen in rats treated with saline (Figure 2A). The histopathological characteristics of the SA group were not significantly different from those of the AG group. AG treatment significantly attenuated bleomycin-induced fibrotic lesions and collagen fiber accumulation in rat lungs in a dose-dependent manner. To confirm the effect of AG on the histopathological change of bleomycin-induced pulmonary fibrosis, the overall grades of the fibrotic changes of the lungs were performed by Ashcroft score (Figure 2C). The score of the BLM+AG group was significantly lower than that of the BLM group (p < 0.01).

Collagen deposition in lung tissues was assessed by measuring the hydroxyproline content. Compared with the SA group, hydroxyproline content was significantly increased in the BLM group after bleomycin infusion. The increased hydroxyproline content in rat lungs was decreased dose-dependently with AG administration (p < 0.05; Figure 2D). However, no significant differences were observed in levels of AGEs, Ashcroft score, and lung hydroxyproline content between the SA and AG groups.

### HSP47 mRNA and protein overexpressions in lung tissues induced by bleomycin are inhibited by AG treatment

**Figure 3:**
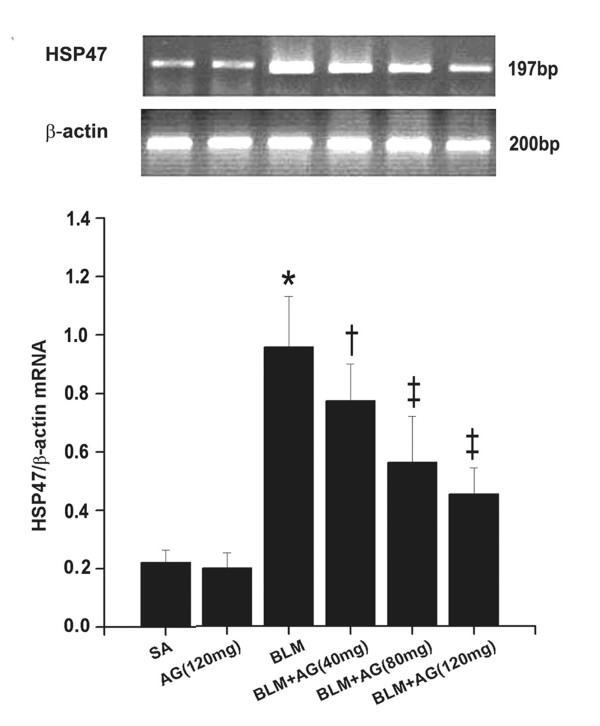
**Effect of AG on bleomycin-induced HSP47 mRNA expression**. The expression of HSP47 mRNA was measured by RT-PCR. The mean ratios of photodensity of HSP47 band to that of β-actin control were shown. Data represent mean ± SD, n = 5 in each group,* p < 0.01 vs SA group; † p < 0.05 vs BLM group; ‡ p < 0.01 vs BLM group.

**Figure 4:**
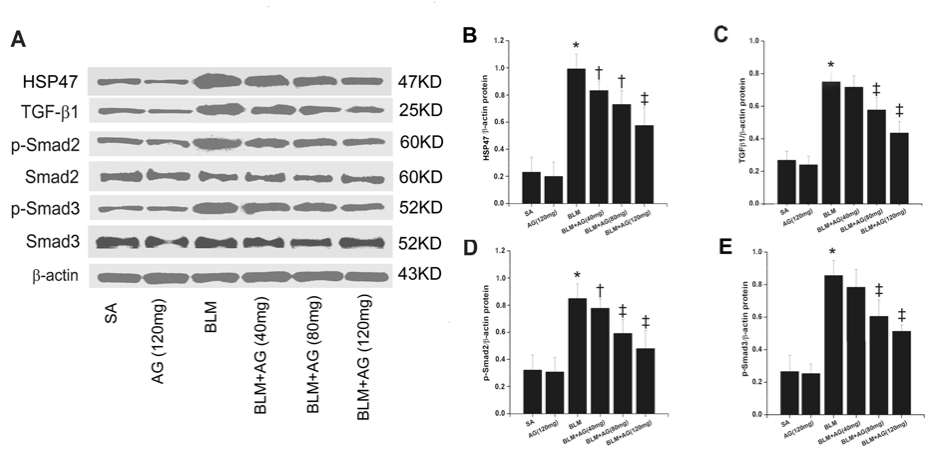
**Effect of AG on HSP47, TGFβ1, p-Smad2, Smad2, p-Smad3 and Smad3 protein after bleomycin instillation**. Western blot was performed to determine the expression levels of target proteins. (A) Representative blotting images of HSP47, TGFβ1, p-Smad2, Smad2, p-Smad3, Smad3, and β-actin were shown. Densitometric analysis of HSP47 (B), TGFβ1 (C), p-Smad2 (D), p-Smad3 (E) protein expression relative to the β-actin control was presented. Data represent mean ± SD, n = 5 in each group, * p < 0.01 vs SA group; † p < 0.05 vs BLM group; ‡ p < 0.01 vs BLM group.

HSP47 mRNA expression in rat lungs was measured by RT-PCR. The expression of HSP47 mRNA in the BLM group was much higher than control rats in the SA group (p < 0.01). AG treatment significantly inhibited BLM-induced HSP47 mRNA expression in lung tissues (p < 0.05, p < 0.01; Figure 3). This inhibitory effect was in a dose-dependent manner. Meanwhile, BLM stimulation significantly increased HSP47 protein expression in rat lungs (p < 0.01), which was inhibited by AG treatment dose-dependently (p < 0.05, p < 0.01; Figure 4A, B). These changes in the Western blot were in accordance with the findings in the RT-PCR study. No significant changes of HSP47 mRNA and protein were revealed in the SA and AG groups.

### TGFβ1, p-Smad2, p-Smad3 protein expressions in lung tissues after bleomycin stimulation are downregulated by AG treatment

As a key factor of pulmonary fibrosis, TGFβ1 was determined by Western blot. BLM significantly increased TGFβ1 protein expression in lung tissues (p < 0.01), which was downregulated by AG treatment dose-dependently (p < 0.01; Figure 4A, C). No significant difference was revealed in TGFβ1 expression level between the SA and AG groups.

Because phosphorylation of Smad signaling by the activated TGFβ1 receptor I is a major step in the initiation of TGFβ1 signal transduction, we further examined whether Smad2 and Smad3 phosphorylation in bleomycin-induced pulmonary fibrosis was changed by AG treatment. Immunoblot analysis showed a marked increase in Smad2 and Smad3 phosphorylation in the BLM lungs over the SA lungs after bleomycin treatment (p < 0.01). AG administration dose-dependently reduced the phosphorylation of Smad2 and Smad3 protein in the bleomycin-induced pulmonary fibrosis (p < 0.05, p < 0.01; Figure 4A, D, E). However, there were no significant changes in total Smad2 and Smad3 expressions among experimental groups (Figure 4A), and no significant differences were observed in Smad2 and Smad3 phosphorylation between the SA group and AG group.

## Discussion

In the present study, BLM stimulation markedly increased the level of AGEs in lung tissues as well as lung hydroxyproline content and fibrosis score, which were inhibited with treatment of AG, an AGE formation inhibitor, in a dose-dependent manner. Further, AG treatment also decreased BLM-induced HSP47 expression, downregulated TGFβ1, p-Smad2 and p-Smad3 expressions, and subsequently attenuated BLM-induced pulmonary fibrosis. From these findings, we conclude that AGEs may play an important role in pulmonary fibrosis induced by BLM, which may be involved in its potentially regulatory effects on HSP47 expression and TGFβ/Smads signaling pathway.

Prior studies have strongly evidenced the positive roles of AGEs in the process of fibrogenesis. Huang et al and Lee et al reported AGE dose- and time-dependently increased collagen production and connective tissue growth factor (CTGF) mRNA and protein expression in NRK-49F (normal rat kidney fibroblast) cells [21,22]. In human foreskin fibroblasts, Lohwasser et al found AGE incubation could increase CTGF, TGF-β1, and procollagen-alpha1 (I) mRNA [23]. Futher more, AGE treatment significantly increased fibronectin and type IV collagen accumulation in renal glomeruli, and also markedly induced renal TGF-β1 and CTGF expression in rats [24]. These *in-vitro *and *in-vivo *experimental studies indicate AGEs could be an effective stimulator in fibrogenesis. In the present study, our data confirm AGEs accumulation is paralleled with the progression of BLM-induced pulmonary fibrosis assessed by lung hydroxyproline assay and fibrotic scoring, and blockade of AGE formation by AG treatment significantly attenuates BLM-induced pulmonary fibrosis, which supports the participation of AGE formation in this process.

As excessive deposition of ECM may contribute to pulmonary fibrosis [1] and collagens are the major fibrous proteins in ECM, we considered AGEs could have effects on collagen synthesis within the process of pulmonary fibrosis. HSP47, as a specific collagen molecular chaperone, was reported to be correlated well with collagen deposition in both animal and human studies [25-27], which suggests an important role of HSP47 in increased deposition of collagens during the progression of fibrotic diseases. Moreover, recent researches by Hagiwara and his colleagues reported that inhibition of HSP47 by antisense oligodeoxynucleotides significantly suppressed the production of collagen and subsequently attenuated pulomonary fibrosis in bleomycin-, lipopolysaccharide- and paraquat-induced pulmonary fibrosis in rats [28-30]. These findings further demonstrate a key role of HSP47 in collagen synthesis during the course of pulmonary fibrosis. The present results show overexpression of HSP47 induced by BLM is dose-dependently inhibited by AG treatment, which indicates that AGE formation may participate in BLM-stimulated pulmonary fibrosis at least partly through upregulation of HSP47 expression, and HSP47 may be a critical target factor of AGEs in BLM-induced pulmonary fibrosis. But so far very little is known about the underlying molecular mechanism by which AGE formation modulates HSP47 expression in BLM-stimulated pulmonary fibrosis.

It has been well-documented that TGFβ1 appears to be the predominant isoform of TGFβs involved in pulmonary fibrosis, which exerts its profibrotic effects through chemoattraction and stimulation of fibroblasts to express growth factors and extracellular matrix components [8]. Several reporters demonstrated TGFβ1, as a major regulator, stimulated HSP47 expression, in parallel with collagen production [26,31-33]. Simultaneously, as was also reported, AGEs could increase both TGFβ1 and HSP47 expression in cultured mesangial cells [6]. Although there are no direct evidences to determine whether TGFβ signaling contributes to AGE induction of HSP47 expression, Li and his colleagues reported AGE induced a rapid Smad2 and Smad3 nuclear translocation and phosphorylation by normal rat tubular epithelial cells, glomerular mesangial cells, and vascular smooth muscle cells in a dose- and time-dependent manner, which was mediated by TGFβ signaling pathway [34], and Ohashi et al further found mesangial cells transfected with Smad1-antisense oligomers showed much less expression of HSP47 and type IV collagen transcripts after AGE stimulation than those with control oligomers [6]. These studies indicate TGFβ/smads might play an important role in the process of AGE-induced HSP47 expression. So, we hypothesised the potentially regulatory effect of AGEs on BLM-induced HSP47 expression was involved in TGFβ/smads pathway. In our study, through inhibition of AGE formation in BLM-induced lung fibrosis by AG treatment, the expressions of TGFβ1, p-Smad2 and p-Smad3 were all downregulated dose-dependently, suggesting TGFβ/Smads signaling pathway probably plays a role in AGE-regulated HSP47 expression induced by BLM, although this link still needs more evidences to confirm.

Taken together, our results demonstrate AGE formation contributes to BLM-stimulated lung fibrosis, and HSP47 may be a potential target factor of AGEs. Blockade of AGE formatioin by AG treatment attenuates BLM-induced HSP47 overexpression, probably through inhibition of TGFβ1/Smad2/Smad3 signaling pathway, which suggests for the first time that AGEs may participate in the process of BLM-induced pulmonary fibrosis, at least partly implicated in TGFβ/Smads-HSP47 pathway. The further study should focus on whether the contribution of AGEs to the lung fibrosis is involved in its receptor, the receptor of AGEs (RAGE). In recent studies, loss of RAGE was observed in the lungs of IPF patients and bleomycin- or asbestos-treated rats [35,36]. In addition, RAGE-null mice developed more severe pulmonary fibrosis than wild-type controls [35], indicative of a protective role of RAGE in lung fibrosis. However, He et al demonstrated that RAGE contributed to bleomycin-induced lung fibrosis through epithelial-mesenchymal transition and profibrotic cytokine production [37]. It can be seen the role of RAGE in pulmonary fibrosis needs further determination.

## Conclusion

AGEs are complex products of nonenzymatic glycation, with links to fibrotic lesions in various disorders. Our findings firstly demonstrate AGE formation may participate in BLM-induced pulmonary fibrosis, and TGFβ/Smads-HSP47 pathway is probably implicated in this process, although more investigations are needed to confirm this mechanism. Moreover, the inhibitory effect of AG on HSP47 expression and TGFβ/smads signaling pathway in BLM-induced pulmonary fibrosis, is supposed to be a beneficial supplement for more understanding of the protective role of AG in BLM-induced pulmonary fibrosis.

## Abbreviations

*AG*: aminoguanidine; *AGE(s)*: advanced glycation end product(s); *BLM*: bleomycin; *CTGF*: connective tissue growth factor; *DW*: distilled water; *ECM*: Extracellular matrix; *ELISA*: Enzyme-Linked ImmunoSorbent Assay; *HSP47*: heat shock protein 47; *p-Smad2/3*: phosphorylated-Smad2/Smad3; *RAGE*: receptor of advanced glycation end products; *RT-PCR*: Reverse Transcriptase-Polymerase Chain Reaction; *TGFβ1*: transforming growth factor β1; *SA*: saline; *SD*: standard deviation.

## Competing interests

The authors declare that they have no competing interests.

## Authors' contributions

LC and TW drafted the manuscript, and LC carried out the data analysis. FW was responsible for the design of the original study. LC, TW and DL participated in the design of animal experiment. LC, XW, BS, JL, LL and YC carried out the animal experiment. LC, TW, SZ and DX carried out the fibrosis score, RT-PCR, Western blot, ELISA, Masson stain and hydroxyproline content assays.
